# Sex, military occupation and rank are associated with risk of anterior cruciate ligament injury in tactical-athletes

**DOI:** 10.1136/bmjmilitary-2021-002059

**Published:** 2022-02-14

**Authors:** Aubrey D Aguero, J J Irrgang, A J MacGregor, S D Rothenberger, J M Hart, J J Fraser

**Affiliations:** 1 Physical Therapy Department, School of Health & Rehabilitation Sciences, University of Pittsburgh, Pittsburgh, Pennsylvania, USA; 2 Naval Medical Leader & Professional Development Command, US Navy Bureau of Medicine and Surgery, Falls Church, Virginia, USA; 3 Operational Readiness & Health Directorate, Naval Health Research Center, San Diego, California, USA; 4 Department of Medicine, University of Pittsburgh School of Medicine, Pittsburgh, Pennsylvania, USA; 5 Department of Orthopaedic Surgery, University of Virginia School of Medicine, Charlottesville, Virginia, USA

**Keywords:** knee, occupational & industrial medicine, orthopaedic sports trauma, sports medicine, epidemiology, musculoskeletal disorders

## Abstract

**Introduction:**

Anterior cruciate ligament (ACL) injury is common within the US military and represents a significant loss to readiness. Since recent changes to operational tempo, there has not been an analysis of ACL injury risk. The aim of this retrospective cohort study was to evaluate military occupation, sex, rank and branch of service on ACL injury risk in the US military from 2006 to 2018.

**Methods:**

The Defense Medical Epidemiology Database was queried for the number of US tactical athletes with International Classification of Diseases diagnosis codes 717.83 (old disruption of ACL), 844.2 (sprain of knee cruciate ligament), M23.61 (other spontaneous disruption of ACL) and S83.51 (sprain of ACL of knee) on their initial encounter. Relative risk and χ^2^ statistics were calculated to assess sex and military occupation effects on ACL injury. A multivariable negative binomial regression model evaluated changes in ACL injury incidence with respect to sex, branch of service and rank.

**Results:**

The study period displayed a significant decrease in the ACL injury rate at 0.18 cases per 1000 person-years or relative decrease of 4.08% each year (p<0.001) after averaging over the main and interactive effects of sex, rank and branch of service. The interaction effect of time with sex indicated a steeper decline in the incidence in men as compared with women. The risk of ACL injury by sex was modified by rank. The incidence among military personnel varied by occupation.

**Conclusion:**

Despite the decline among tactical athletes over time, rates of ACL injury remain much higher than the general US population. Sex, rank, branch of service and military occupation were found to be risk factors for ACL injury. It is critical for policy makers to understand the salient risk factors for ACL injury to guide proactive measures to prevent injury.

**Key messages**

- Anterior cruciate ligament (ACL) injury is a known command readiness issue in the military, and there is recent evidence of this within subpopulations of the military.
- Sex, rank, branch of service and military occupation have been found to be risk factors for ACL injury.
- There was a 4.08% decrease in the incidence of ACL injuries between the years 2006 and 2018.
- The rate of decrease was higher in men when considering rank and branch of service.
- The relationship of ACL injury incidence and sex was modified by rank.
- It is critical for policy makers to understand the salient risk factors for ACL injury to guide appropriate prophylactic measures to prevent injury.

**Questions for future research**

- Does integration of military rehabilitation specialists into patient-centred medical homes and operational units affect anterior cruciate ligament (ACL) injury risk, rehabilitation volume and return to duty rates?
- Does integration of female tactical athletes in combat arms affect ACL injury incidence?
- What are the granular modifiable and non-modifiable intrinisic risk factors that mediate ACL injury outcomes in military tactical athlete?

## Introduction

Anterior cruciate ligament (ACL) injury in military members(also referred to as tactical athletes) is estimated to be 10 times that of the civilian population,^1 2^ which is comparable to the rate of injury in professional or elite athletes.^3^ Previous epidemiological studies of ACL injury in the military population have focused on specific communities such as the US Naval Special Warfare community^4^ and midshipmen at the US Naval Academy^5^; however, these groups are highly active and not representative of the typical military member. In the first military-wide population study assessing ACL injury, Owens *et al* 1 reported the incidence in the US military was 6.6 per 1000 person-years from 1993 to 2003. The burden of these injuries threaten command readiness,^6^ especially during periods of increased operational requirements. Since the end of the study period investigated by Owens *et al* 1 coincided with the beginning of the conflicts Operation Enduring Freedom (OEF) in 2001 and Operation Iraqi Freedom (OIF) in 2003, an updated estimate of ACL injury incidence in the US military is needed.

Each service branch comprises an array of diverse military occupations, each of which have unique physical requirements and hazards that may influence injury rates. An overview of military occupational specialties can be viewed at https://bitly/MilOcc. Furthermore, rank may also be a factor due to changes in type and amount of exposure to potential hazards throughout a military career. While Antosh *et al* 7 investigated rank as a factor for return to duty after ACL reconstruction, there is a dearth of evidence pertaining to this as a contributing factor to ACL injuries in military tactical athletes.

Finally, rescission of the ‘*1994 Direct Ground Combat Definition and Assignment Rule*’ in January 2013^8^ fostered the integration of women into military occupations that were previously closed to them, such as combat arms. The Secretary of Defense issued a memorandum to direct all branches to execute their plans to fully implement women into all occupational specialties no later than 1 April 2016.^9^ Since this time, it is unclear how this policy has affected the incidence of ACL injury by sex within occupational specialties. Due to changes in force composition and operational requirements, an updated assessment of the factors associated with ACL injury risk in the tactical athlete is warranted. Therefore, the aim of this epidemiological retrospective cohort study was to evaluate the effects of military occupation, sex, rank and service branch on the risk of ACL injury in the US military from 2006 to 2018.

## Methods

A population-based epidemiological retrospective cohort study of all tactical athletes in the US Armed Forces was performed to assess the risk of sex, rank, service branch and military occupation on the incidence of ACL injury. The Defense Medical Epidemiological Database ((DMED), Defense Health Agency, Falls Church, Virginia, USA, https://bit.ly/DHADMEDv5) was used to identify relevant healthcare encounters. This database provides aggregated data for International Classification of Diseases (ICD)-9 and ICD-10 codes and de-identified patient characteristics, including sex, rank, categories of military occupations and service branch for all active and reserve tactical athletes. The database does not include any personal identifiable or personal health information and has been used previously for epidemiological study of lower extremity injury in the military.^1 10 11^

The database was queried for the number of distinct patients with a primary diagnosis of ACL injury (717.83 (old disruption of ACL), 844.2 (sprain of knee cruciate ligament), M23.61 (other spontaneous disruption of ACL) and S83.51 (sprain of ACL of knee)) on their initial encounter from 2006 to 2018. Individuals with repeat visits for the same diagnosis were only counted once in all analyses.

Cumulative incidence of ACL injury in male and female tactical athletes, enlisted members and officers, in each service branch (Army, Navy, Marine Corps and Air Force) and occupational category (enlisted specialties: special operations forces, mechanised/armour, artillery/gunnery, aviation, engineers, maintenance, administration, intelligence and communication, logistics and maritime/naval specialties; officer specialties: aviation, engineering and maintenance, administration, operations and intelligence, logistics and services) were calculated. Since military end strength fluctuates annually due to attrition and recruitment of replacements,^12^ the population at risk was a dynamic cohort. Incidence was calculated from the sum of ACL injuries and population at risk in the 13-year study epoch. Relative risk (RR) point estimates and 95% CIs, risk difference, attributable risk (AR), number needed to harm (NNH) and χ^2^ statistics were calculated to assess the effects of sex and military occupational category. The preceeding calculations were performed using Microsoft Excel for Mac 2016 (Microsoft, Redmond, Washington, USA).

A multivariable negative binomial regression was performed to evaluate time trends with respect to ACL injury incidence and included the factors of sex, rank and branch of service. Average marginal effects of predictor variables in the model were estimated for ease of interpretation. Due to overdispersion of the data indicated by the likelihood ratio test demonstrating that alpha is significantly different from zero, the negative binomial model was chosen over the Poisson regression model.^13^ The regression analysis was performed using Stata 16 software (StataCorp, College Station, Texas, USA).

Male tactical athletes were the reference group in the assessment of sex. Enlisted personnel served the reference group for rank due to the greater disease and non-battle injuries in this group compared with commissioned officers.^14^ Enlisted infantry and ground and naval gunfire officer groups were the reference in the assessment of occupational risk due to relatively higher physical requirements and organisational prioritisation of these occupational categories.^11 15^ Finally, the army was the reference group for service branch due to the population size and injury rates compared with the other services.^16^ The level of significance was p<0.05 for all analyses, and no adjustments were made for multiplicity. RR point estimates were considered statistically significant if CIs did not cross 1.00.

## Results

From 2006 to 2018, 59 555 enlisted male officers sustained ACL injuries (4.8 per 1000 person-years) and 8350 enlisted female officers sustained ACL injuries (3.9 per 1000 person-years) for a total of 67 905 enlisted member ACL injuries across the services (4.6 per 1000 person-years). During the same time period, 9983 male officers sustained ACL injuries (4.0 per 1000 person-years) and 2198 female officers sustained ACL injuries (4.5 per 1000 person-years) for a total of 12 181 officer ACL injuries across the services (4.1 per 1000 person-years). Tables 1 and 2 display the counts and incidence of ACL injury. Online supplemental tables 1–4 detail the ACL injury counts, population at risk and injury rates in male and female enlisted members and officers.

10.1136/bmjmilitary-2021-002059.supp1 Supplementary data

10.1136/bmjmilitary-2021-002059.supp2 Supplementary data

10.1136/bmjmilitary-2021-002059.supp3 Supplementary data

10.1136/bmjmilitary-2021-002059.supp4 Supplementary data

**Table 1:** Count and incidence of anterior cruciate ligament sprains among officers in the US Armed Forces, 2006–2018

Officer specialty	Ground and naval gunfire			Aviation			Engineering and maintenance			Administration			Operations and intelligence			Logistics			Services			Total		
Injuries (n)																								
	M	F	Total	M	F	Total	M	F	Total	M	F	Total	M	F	Total	M	F	Total	M	F	Total	M	F	Total
Army	1110	24	1134	442	28	470	736	121	857	276	116	392	428	120	548	430	122	552	785	364	1149	4328	913	5241
Navy	388	49	437	435	37	472	279	23	302	80	23	103	151	32	183	100	12	112	351	200	551	2017	444	2461
Air Force	51	2	53	693	42	735	397	72	469	171	81	252	356	124	480	266	87	353	494	285	779	2673	742	3415
Marines	201	2	203	197	13	210	102	9	111	77	15	92	106	6	112	124	22	146	16	3	19	965	99	1064
Total	1750	77	1827	1767	120	1887	1514	225	1739	604	235	839	1041	282	1323	920	243	1163	1646	852	2498	9983	2198	12181
**Incidence (per 1000 person-years)**																								
Army	4.5	7.1	4.5	4.0	4.5	4.1	4.6	4.6	4.6	4.0	4.4	4.1	4.6	6.7	4.9	4.7	4.7	4.7	3.9	4.3	4.0	4.3	4.7	4.4
Navy	3.4	3.5	3.4	3.5	4.5	3.5	3.8	5.2	3.9	3.0	3.2	3.0	4.0	5.0	4.1	3.6	2.5	3.4	3.3	3.6	3.4	3.5	3.9	3.6
Air Force	5.0	1.2	4.5	3.4	3.8	3.5	4.4	5.0	4.5	4.2	4.8	4.4	3.7	5.4	4.1	5.4	6.6	5.7	3.9	3.9	3.9	4.0	4.6	4.1
Marines	3.9	3.2	3.9	3.7	7.9	3.8	3.5	5.8	3.6	3.0	3.8	3.1	4.3	3.4	4.2	3.9	5.3	4.0	2.4	3.2	2.5	3.8	5.7	3.9
Total	4.1	3.9	4.1	3.6	4.4	3.7	4.3	4.8	4.3	3.7	4.3	3.9	4.1	5.8	4.4	4.6	5.0	4.7	3.7	3.9	3.8	4.0	4.5	4.1
Occupation codes	O205: Ground and Naval Arms; O206: Missiles			O201: Fixed-Wing Fighter/Bomber Pilots; O202: Other Fixed-Wing Pilots; O203: Helicopter Pilots; O204: Aircraft Crews			OFF4: Engineering and Maintenance Officers			OFF1: General Officers and Executives, N.E.C.; OFF7: Administrators			O207: Operations Staff; O301: General Intelligence; O302: Communications Intelligence; O303: Counterintelligence			OFF8: Supply, Procurement and Allied Officers			OFF5: Scientists and Professionals; OFF6: Healthcare Officers			All officer specialties		

F, female; M, male.

**Table 2:** Count and incidence of anterior cruciate ligament sprains among enlisted members in the US Armed Forces, 2006–2018

Enlisted specialty	Special operations forces			Infantry			Mechanised/Armour			Artillery/Gunnery			Aviation			Engineers			Maintainance			Administration, intelligence and communication			Logistics			Maritime/Naval specialties			Total		
Injuries (n)																																	
	M	F	Total	M	F	Total	M	F	Total	M	F	Total	M	F	Total	M	F	Total	M	F	Total	M	F	Total	M	F	Total	M	F	Total	M	F	Total
Army	513	**	513	4445	**	4445	543	**	543	1474	80	1554	**	**	**	811	18	829	5957	537	6494	6097	1209	7306	3539	660	4199	29	1	30	24 634	3406	28 040
Navy	133	**	133	60	**	60	5	**	5	139	28	167	292	24	316	**	**	**	6570	788	7358	2256	445	2701	948	173	1121	518	77	595	12 512	1953	14 465
Air Force	**	**	**	**	**	**	**	**	**	**	**	**	264	21	285	**	**	**	5464	314	5778	3264	847	4111	1699	332	2031	**	**	**	12 177	2252	14 429
Marines	**	**	**	1855	**	1855	180	**	180	242	**	242	97	2	99	229	14	243	3179	152	3331	2609	290	2899	1247	100	1347	**	**	**	10 232	739	10 971
Total	646	**	646	6360	**	6360	728	**	728	1855	108	1963	653	47	700	1040	32	1072	21170	1791	22961	14226	2791	17017	7433	1265	8698	547	78	625	59 555	8350	67 905
Cumulative incidence (per 1000 person-years)																																	
Army	6.3	**	6.3	5.5	**	5.5	5.4	**	5.4	5.8	7.1	5.8	**	**	**	5.7	5.9	5.7	5.5	5.1	5.5	5.4	3.8	5.1	6.0	5.0	5.8	5.3	1.5	4.8	5.2	4.6	5.1
Navy	5.9	**	5.9	4.5	**	4.5	3.2	**	3.2	4.0	4.7	4.1	5.2	4.4	5.1	**	**	**	4.4	3.6	4.3	4.5	2.8	4.1	4.7	3.3	4.4	4.6	2.8	4.2	4.3	3.3	4.1
Air Force	**	**	**	**	**	**	**	**	**	**	**	**	4.7	6.3	4.8	**	**	**	4.7	3.7	4.6	5.3	3.1	4.6	5.0	4.5	4.9	**	**	**	4.4	3.4	4.2
Marines	**	**	**	4.8	**	4.8	5.9	**	5.9	5.6	**	5.6	4.5	2.7	4.4	4.6	6.4	4.7	5.9	5.1	5.8	6.0	4.1	5.7	5.5	4.0	5.3	**	**	**	5.0	4.6	5.0
Total	6.2	**	6.2	5.3	**	5.3	5.5	**	5.5	5.6	6.3	5.6	4.9	4.9	4.9	5.5	6.1	5.5	4.9	4.1	4.9	5.3	3.4	4.9	5.4	4.5	5.3	4.6	2.8	4.3	4.8	3.9	4.6
Occupation codes	E011: Special Forces			E010: Infantry, General			Occupation: E020: Armour and Amphibious, General			E041: Artillery and Gunnery; E042: Rocket Artillery; E043: Missile Artillery, Operating Crew			E050: Air Crew, General; E051: Pilots and Navigators			E030: Combat Engineering, General			ENL7: Craftsworkers; ENL1: Electronic Equipment Repairers; ENL6: Electrical/Mechanical Equipment Repairers			ENL2: Communications and Intelligence Specialist; ENL5: Functional Support and Administration			ENL8: Service and Supply Handlers			E062: Small Boat Operators; E063: Seamanship, General; E060: Boatswains			All enlisted specialities		

** no tactical-athletes in the population at-risk for these categories

F, female; M, male.

**Figure 1:**
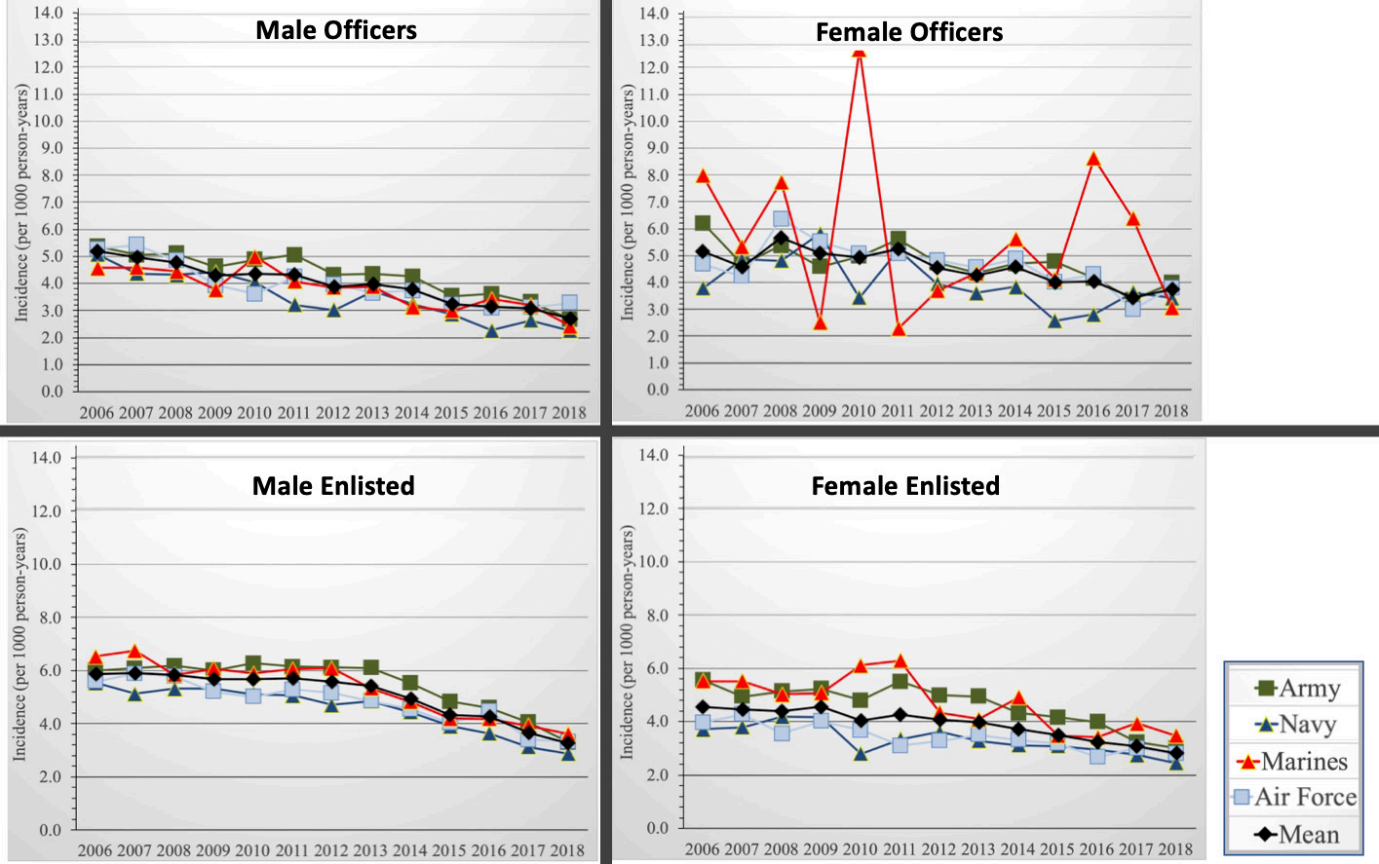
Anterior cruciate ligament injury incidence among male and female enlisted members and officers, US Armed Forces, 2006–2018.

ACL injury rates decreased over time during the study epoch (Figure 1). Male officer incidence decreased from 5.2 (95% CI: 4.9 to 5.5) to 2.7 (95% CI: 2.5 to 2.9) per 1000 person-years, female officer incidence decreased from 5.1 (95% CI: 4.3 to 5.9) to 3.7 (95% CI: 3.1 to 4.3) per 1000 person-years, enlisted male incidence decreased from 5.9 (95% CI: 5.7 to 6.1) to 3.3 (95% CI: 3.2 to 3.4) per 1000 person-years and enlisted female incidence decreased from 4.5 (95% CI: 4.2 to 4.8) to 2.8 (95% CI: 2.6 to 3.0) per 1000 person-years. Of note, the increase in incidence seen by female officers in the US Marines during 2010 and 2016 may potentially coincide with the changes in physical fitness standards for the Combat Fitness Test in the US Marine Corps. There is a plausible influence that the changes in standards may have resulted in overengagement in risk-taking activity to meet these standards.

Table 3 reports the risk of ACL injury by sex, with females referenced to males within their respective rank (officer vs enlisted) and occupation. The risk of ACL injury in female officers was 1.14 times that of their male colleagues (95% CI: 1.09 to 1.19; AR: 12.0%; NNH: 1836; p<0.01) and was greater in aviation, administration and operations and intelligence occupations (RR: 1.16–1.39; AR: 14.0%–28.1%; NNH: 617–1650; p<0.05) compared with male officers. The risk of ACL injury in female enlisted members was 0.82 times that of their male colleagues (95% CI: 0.80 to 0.83; AR: −22.7%; NNH: −1134; p<0.01) and was lower in maintenance, administration, intelligence and communication, logistics and maritime/naval specialties compared with male enlisted members (RR: 0.61–0.83; AR: −64% to −20.9%; NNH: −1173 to −523; p<0.01).

**Table 3:** ACL injury risk by sex in members of the US Armed Forces, 2006–2018

Enlisted specialty	Artillery/Gunnery	Aviation	Engineers	Maintenance	Administration, intelligence and communication	Logistics	Maritime/Naval specialties	Total
RR (95% CI)	1.13 (0.93 to 1.37)	1.01 (0.75 to 1.36)	1.12 (0.79 to 1.58)	0.83 (0.79 to 0.87)	0.64 (0.61 to 0.37)	0.82 (0.77 to 0.87)	0.61 (0.48 to 0.77)	0.82 (0.80 to 0.83)
Risk difference (per 1000 person-years)	0.7	0.1	0.6	−0.9	−1.9	−1.0	−1.8	−0.9
AR (%)	11.3	1.2	10.4	−20.9	−56.2	−21.8	−64	−22.7
P value	0.22	0.94	0.54	**<**0.001	**<**0.001	**<**0.001	**<**0.001	**<**0.001
NNH	547	16 600	1586	−1173	−523	−1027	−558	−1134
**Officer specialty**	**Ground/Naval gunfire**	**Aviation**	**Engineering and maintenance**	**Administration**	**Operations and intelligence**	**Logistics**	**Services**	**Total**
RR (95% CI)	0.95 (0.75 to 1.19)	1.22 (1.01 to 1.47)	1.12 (0.98 to 1.29)	1.16 (1.00 to 1.35)	1.39 (1.22 to 1.59)	1.10 (0.96 to 1.27)	1.05 (0.97 to 1.14)	1.14 (1.09 to 1.19)
Risk difference (per 1000 person-years)	−0.2	0.8	0.5	0.6	1.6	0.5	0.2	0.5
AR (%)	−5.5	18.0	11.1	14	28.1	9.1	5.1	12.0
P value	0.64	0.03	0.10	0.05	**<**0.001	0.19	0.21	**<**0.001
NNH	−4631	1258	1875	1650	617	2185	4924	1836

Female service members referenced to male members.

AR, attributable risk; NNH, number needed to harm; RR, relative risk.

Results of the assessment of occupation on ACL injury risk are detailed in Table 4. Enlisted personnel in aviation, maintenance, administration, intelligence, communication and maritime/naval specialties had a 0.81–0.93 lower risk compared with infantry (AR: −23.5% to −8.0%; NNH: −2576 to −1002; p<0.05), and Special Operation Forces and Artillery/Gunnery occupations had a 1.07–1.19 greater risk compared with infantry (AR: 6.3%–15.8%; NNH: 1013–2827; p<0.01). Aviation officers and services officers had a 0.89–0.92 lower risk of ACL injury compared with ground and naval gunfire officers (AR: −12.9% to −8.2%, NNH: −2127 to −3187, p<0.01), and logistics officers had a 1.13 greater risk compared with ground and naval gunfire officers (AR: 11.8%; NNH: 1808, p<0.01).

**Table 4:** ACL injury risk by occupation in members of the US Armed Forces, 2006–2018

Enlisted specialty*	Special operation forces	Mechanised/Armour	Artillery/Gunnery	Aviation	Engineers	Maintenance	Administration, intelligence and communication	Logistics	Maritime/Naval specialties
RR (95% CI)	1.19 (1.10 to 1.29)	1.05 (0.97 to 1.13)	1.07 (1.01 to 1.12)	0.93 (0.86 to 1.00)	1.04 (0.98 to 1.11)	0.93 (0.90 to 0.95)	0.93 (0.90 to 0.95)	1.00 (0.97 to 1.04)	0.81 (0.75 to 0.88)
Risk difference (per 1000 person-years)	1.0	0.3	0.4	−0.4	0.2	−0.4	−0.4	0.0	−1.0
AR (%)	15.8	4.6	6.3	−8.0	3.9	−8.0	−8.0	−0.2	−23.5
P value	**<**0.001	0.22	0.01	0.05	0.22	**<**0.001	**<**0.001	0.88	**<**0.001
NNH	1013	3915	2827	−2576	4635	−2564	−2571	−78292	−1002
**Officer specialty†**	**Aviation officers**	**Engineering and maintenance**	**Administration**	**Operations and intelligence**	**Logistics**	**Services**			
RR (95% CI)	0.89 (0.83 to 0.94)	1.05 (0.99 to 1.12)	0.94 (0.87 to 1.02	1.07 (1.00 to 1.15)	1.13 (1.05 to 1.22)	0.92 (0.87 to 0.98)			
Risk difference (per 1000 person-years)	−0.5	0.2	−0.3	0.3	0.6	−0.3			
AR (%)	−12.9	5.0	−6.5	6.5	11.8	−8.2			
P value	**<**0.001	0.12	0.13	0.06	**<**0.001	0.01			
NNH	−2127	4581	−3984	3479	1808	−3187			

*Contrasted to enlisted infantry.

†Referenced to ground and naval gunfire officers.

AR, attributable risk; NNH, number needed to harm; RR, relative risk.

The multivariable negative binomial regression demonstrated significant effects of time, sex, an interaction effect of sex with time, rank and branch of service on the incidence of ACL injury. Through calculation of the average marginal effect, the decrease in ACL injury incidence is 0.18 cases per 1000 person-years (95% CI: 0.16 to 0.20 per 1000 person-years, p<0.01), after averaging over the main and interactive effects of sex, rank and branch of service. This decrease is a 4.08% relative reduction in the injury rate per year (95% CI: 3.56% to 4.60%, p<0.01). Officers had a 0.89 times lower rate of ACL injury (95% CI: 0.86 to 0.93, p<0.01) compared with enlisted personnel. The US Navy and US Air Force demonstrated 0.79 times (95% CI: 0.75 to 0.84, p<0.01) and 0.87 times (95% CI: 0.82 to 0.91, p<0.01) the rate of the US Army, respectively, while the US Marine Corps was not statistically different compared with the US Army (0.96, 95% CI: 0.91 to 1.02, p=0.17).

**Figure 2:**
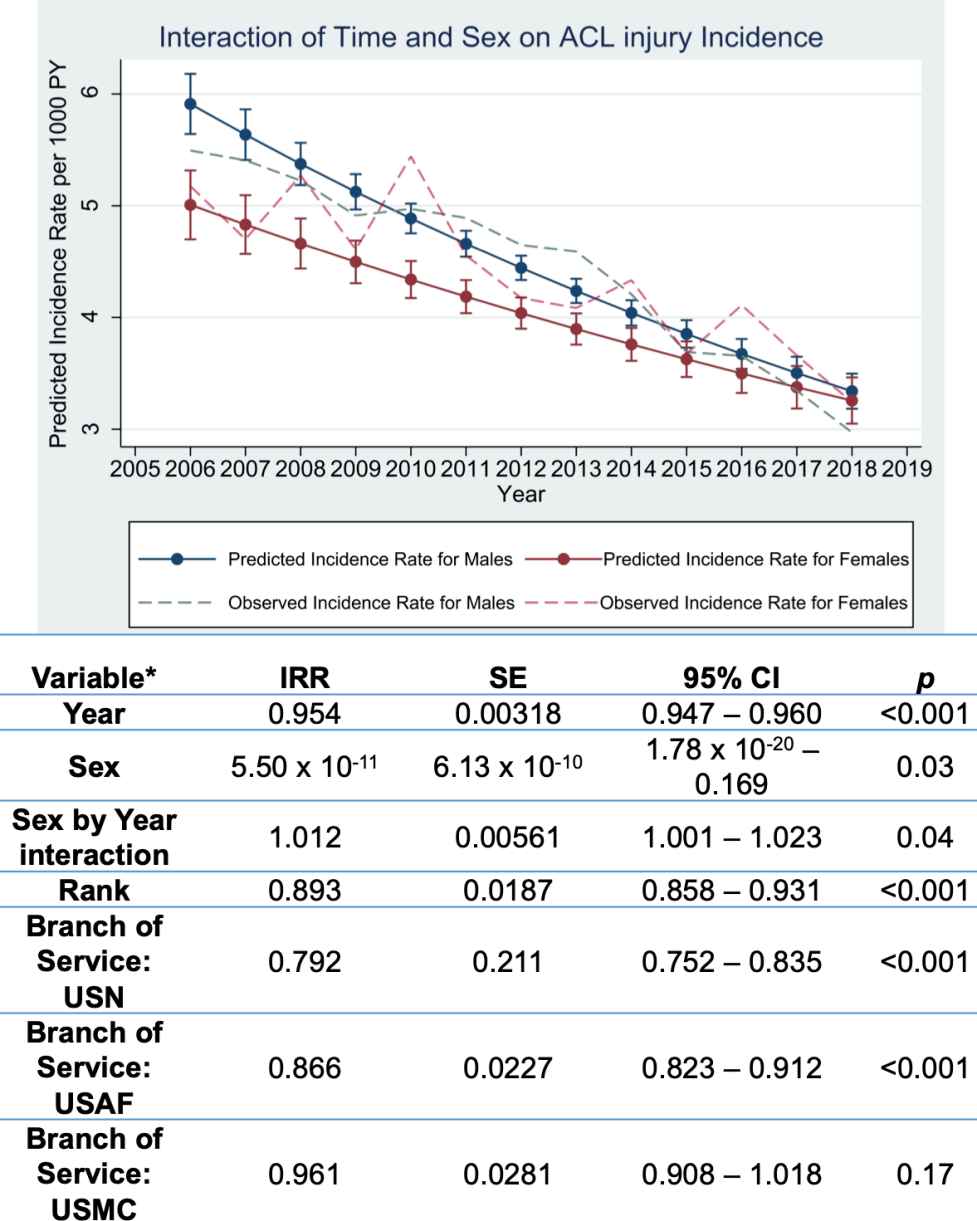
Negative binomial regression model for incident rate ratio (IRR) estimates of ACL injury over time, adjusted for sex, rank and branch of service, in members of the US Armed Forces, 2006–2018. USAF, United States Air Force; USMC, United States Marine Corps; USN, United States Navy; SE, standard error; CI, confidence interval.

Figure 2 depicts the interaction effect of time and sex on the outcome of ACL injury incidence. Through calculation of the average marginal effect for year, a significant difference was found in the rate of decrease between male and female officers (p<0.01). The decrease in incidence in male officers was greater compared with female officers, with the difference in the rate of decrease per year at 0.069 cases per 1000 person-years (95% CI: 0.021 to 0.12, p<0.01).

## Discussion

The primary finding of this study was the significant decrease in ACL injury rates over time regardless of sex, rank or branch of service, with injury rates declining at a steeper rate in male compared with female tactical athletes. The findings that ACL injury risk was modified by rank and occupation in male and female tactical athletes are the first to our knowledge to be reported.

### Injury rates over time

The decrease in incidence of ACL injury likely represents a real trend with a potential contributing factor of changes in the operational tempo in the US Armed Forces over the study epoch. This could be plausibly explained by a decreased exposure to hazards with the decline of operational demands. The higher rates of ACL injury at the beginning of this study in 2006 represents a time of increased military operations with a high frequency of multiple deployements involved in OIF and OEF. Activity for these campaigns peaked in 2008,^17^ and the campaigns concluded for OIF in 2011 and OEF in 2014.^18^ Changes in coding may explain a small portion of this change as the ICD-10 coding transition was mandated to occur in October 2015. However, the decline in ACL injury rates occurred prior to this timepoint. While there should be a direct mapping of the codes used in this study, it is unclear how injury data for this study may have been affected.

### Sex and rank

Enlisted members, on average, had higher rates of ACL injury, but the risk of ACL injury by sex demonstrated an effect modification depending on rank. Female officers had a higher risk compared with male officers regardless of occupation. This was contrasted by enlisted females who demonstrated lower risk compared with enlisted males regardless of occupation. The prior population-based study by Owens *et al* 1 did not stratify by rank, which potentially masked risk differences between male and female officers.

The relationship between sex and rank is noteworthy when compared with what is reported in the athlete population. Accounting for the relative numbers of male and female athletes, incidence of ACL injury is higher in female athletes.^19^ Furthermore, when highly active groups of tactical athletes were studied, higher incidence of ACL injury was reported in female tactical athletes.^5^ It was surprising that the relative risk of ACL injury was lower in female enlisted personnel compared with their male counterparts regardless of occupation for this younger group^20^ within the military. This finding, when considered with the injury reduction observed in officers, challenges the assumption that younger, physically active female officers are at higher risk for ACL injury.

One important difference between athletes and tactical athletes is that men and women train side by side in the US military, whereas competitive sports are stratified by sex. While tactical athletes are required to meet age-specific and sex-specific physical fitness testing standards, this is not the case regarding occupational requirements. As women increasingly enter into occupations that were previoulsy closed to them, it is plausible that the findings of sex, rank and military occupation will likely change. Finally, it is possible that young, enlisted males are participating in activities outside of their military occupation that are increasing the risk for ACL injury in this group compared with their female counterparts.

### Occupation and rank

Among officers and enlisted, the risk of ACL injury varied depending on occupation. Exposures to hazards may contribute to the occupational differences observed. This study found that enlisted occupations where vehicles were primarily employed or were more sedentary in nature compared with infantry had lower risk of ACL injury. Aviation, maintenance, administration, intelligence, communication and maritime/naval specialties had significantly lower risk compared with infantry. Occupations where the knee is loaded on uneven surfaces are a known predisposing factor for ACL injury.^21^ Infantry members have higher exposure to hazards that can lead to ACL injury, such as rucking, manoeuvring and training over variable terrain. Similarly, aviation and services officers had a statistically lower risk of ACL injury compared with ground and naval gunfire officers.

Special Operations Forces and Artillery/Gunnery occupations were at a statistically higher risk of ACL injury compared with infantry. The highest risk of ACL injury by occupation found in this study was in the Special Operations Forces; this may be explained by the increased intensity and frequency of tactical training with a correspondingly high level of musculoskeletal injury that is known to occur in this community.^22^ Occupation-specific training and physical activity levels alone are likely not the only important factors driving ACL injury. Risk-taking behaviours that are culturally influenced and vary by occupational communities are likely contributing factors as well.

### Social determinants

ACL injury is not typically self-limiting, and the billed medical encounters used to generate the data in this study are more likely to represent the true incidence. Due to the severity of an acute ACL injury, bias due to healthcare utilisation is not likely to have a large impact on the study results. However, even with ACL injury, barriers to seeking care by tactical athletes should be considered. Fear that future career opportunities may be negatively affected is a concern that may result in under-reporting of injuries.^23^ Additional reasons that may affect reporting include the service member’s perception of the convenience and quality of medical care they will receive.^24^ The cultural environment of the military reinforces the desire to put aside pain associated with an injury to ensure that the mission is completed and to avoid the negative perceptions associated with injury.^24^ It is also plausible that under-reporting may disproportionately affect certain occupations more than others.^25^

### Clinical and research implications

This study highlight important trends of ACL injury in regard to sex, occupation, rank, branch of service and changes over time. Specific hazards and exposures associated with military occupations should be explored in order to mitigate the risks. This is especially critical in communities such as Special Operations Forces, where a relatively smaller number of specialised tactical athletes must perform highly demanding physical tasks that are crucial to mission accomplishment. Surveillance of ACL injury should continue as the percentage of women in previously restricted combat roles grows. It is essential for policymakers to understand the salient factors associated with ACL injury in the military and within subpopulations, so appropriate prophylaxis and injury management can be planned. As rehabilitation specialists across the military continue to be incorporated into patient-centred medical homes and assigned to operational units, the effect on injury risk, rehabilitation and return to duty rates should be investigated.

### Strengths and limitations

The DMED allows for a population-based analysis which provides the best estimation of ACL injury incidence to be captured based on billed medical encounters. This permitted the calculation of sex as a non-modifiable intrinsic factor and exploration of time, rank, branch of service and military occupation as factors for ACL injury. There are also important limitations associated with this study due to inherent constraints associated with DMED. While using initial encounters allowed for the calculation of incidence, this study is also limited in the ability to capture laterality of an injury, and a new injury on the contralateral side may not be counted as such. Salient factors associated with ACL injury that have been identified in tactical athletes are unable to be measured with this database, to include factors such as medical history or body mass index. Finally, a limitation of the diagnosis code 844.2 (sprain of knee cruciate ligament) is the inclusion of posterior cruciate ligament (PCL) injuries. However, PCL injuries are relatively scarce in comparison to ACL injuries and would likely only add minimal bias to the overall results of this study.

## Conclusion

Sex, rank, branch of service and military occupation have been found to be risk factors for ACL injury. There was a statistically significant decrease in the incidence of ACL injuries among tactical athletes in the US Armed Forces between the years 2006 and 2018 at an average rate of 0.18 cases per 1000 person-years or a 4.08% relative reduction each year. The rate of decrease was higher in male tactical athletes when considering rank and branch of service. The relationship of ACL injury incidence and sex was modified by rank. It is plausible that the physical demands and opportunity for exposure within specific military occupations in the enlisted and officer communities may play a role in the differences in ACL injury incidence among occupations reported in this study. Despite the decline in incidence among tactical athletes in the US military over time, the rates of ACL injury still remain higher than the civilian population.

This work was presented at the 63rd Annual Meeting of the Society of Military Orthopaedic Surgeons in Olympic Valley, California, USA, December 2021. The preprint of this manuscript is archived on medRxiv at DOI: 10.1101/2021.09.30.21264383. This study was completed by Dr Aubrey Aguero in partial fulfilment of the academic requirements for the PhD in Rehabilitation Sciences at the University of Pittsburgh.

## Data Availability

All data relevant to the study are included in the article or uploaded as supplementary information. Not applicable.
