# Supplementary material for: Sex, military occupation and rank are associated with risk of anterior cruciate ligament injury in tactical-athletes
Source: BMJ Mil Health. 2022 Feb 14;169(6):535–41. doi: 10.1136/bmjmilitary-2021-002059 (PMC10715491; doi:10.1136/bmjmilitary-2021-002059)
Supplement: Supplementary data [file bmjmilitary-2021-002059supp001.pdf]

Supplemental Table 1: ACL injury counts, population at risk, and injury rates (per 1,000 person-years) by year for male officers

**Counts**	2006	2007	2008	2009	2010	2011	2012	2013	2014	2015	2016	2017	2018	Total
Army	372	360	375	352	384	412	356	360	348	281	278	248	202	4,328
Navy	226	192	189	194	179	144	135	166	146	128	101	117	100	2,017
Air Force	307	300	257	209	193	224	206	188	189	155	148	145	152	2,673
Marines	82	84	84	74	101	85	80	79	62	58	66	62	48	965
Total	987	936	905	829	857	865	777	793	745	622	593	572	502	9,983
**Population**														
Army	69,311	71,314	73,556	76,044	78,737	81,506	82,562	82,654	81,903	79,386	76,947	74,910	75,110	1,003,939
Navy	44,641	44,059	43,828	43,986	44,341	44,732	44,667	44,699	45,016	44,766	44,622	44,270	44,342	577,969
Air Force	58,175	55,391	53,124	53,237	53,485	52,821	52,042	51,692	50,635	48,324	47,944	46,467	46,317	669,654
Marines	17,900	18,280	18,869	19,626	20,223	20,886	20,727	20,296	19,777	19,436	19,307	19,406	19,713	254,446
Total	190,027	189,044	189,378	192,893	196,786	199,944	199,998	199,341	197,330	191,912	188,820	185,053	185,481	2,506,008
**Rate**														
Army	5.4	5.0	5.1	4.6	4.9	5.1	4.3	4.4	4.2	3.5	3.6	3.3	2.7	4.3
Navy	5.1	4.4	4.3	4.4	4.0	3.2	3.0	3.7	3.2	2.9	2.3	2.6	2.3	3.5
Air Force	5.3	5.4	4.8	3.9	3.6	4.2	4.0	3.6	3.7	3.2	3.1	3.1	3.3	4.0
Marines	4.6	4.6	4.5	3.8	5.0	4.1	3.9	3.9	3.1	3.0	3.4	3.2	2.4	3.8
Total	5.2	5.0	4.8	4.3	4.4	4.3	3.9	4.0	3.8	3.2	3.1	3.1	2.7	4.0
