# Supplementary material for: Sex, military occupation and rank are associated with risk of anterior cruciate ligament injury in tactical-athletes
Source: BMJ Mil Health. 2022 Feb 14;169(6):535–41. doi: 10.1136/bmjmilitary-2021-002059 (PMC10715491; doi:10.1136/bmjmilitary-2021-002059)
Supplement: Supplementary data [file bmjmilitary-2021-002059supp002.pdf]

Supplemental Table 2: ACL injury counts, population at risk, and injury rates (per 1,000 person-years) by year for female officers.

**Counts**	2006	2007	2008	2009	2010	2011	2012	2013	2014	2015	2016	2017	2018	Total
Army	78	60	72	65	74	87	75	70	76	76	65	52	63	913
Navy	29	37	37	46	28	44	34	32	35	24	27	36	35	444
Air Force	61	52	75	66	62	62	59	57	61	49	53	37	48	742
Marines	9	6	9	3	16	3	5	6	8	6	13	10	5	99
Total	177	155	193	180	180	196	173	165	180	155	158	135	151	2,198
**Population**														
Army	12,563	12,858	13,418	14,189	14,876	15,498	15,883	16,059	16,158	15,921	15,666	15,492	15,859	194,440
Navy	7,671	7,612	7,704	7,901	8,141	8,427	8,625	8,869	9,137	9,328	9,639	9,919	10,262	113,235
Air Force	13,032	12,263	11,784	11,967	12,203	12,182	12,233	12,518	12,531	12,165	12,334	12,374	12,623	160,209
Marines	1,127	1,124	1,161	1,201	1,264	1,312	1,354	1,379	1,426	1,454	1,507	1,566	1,639	17,514
Total	34,393	33,857	34,068	35,258	36,483	37,419	38,096	38,825	39,253	38,867	39,145	39,351	40,383	485,398
**Rate**														
Army	6.2	4.7	5.4	4.6	5.0	5.6	4.7	4.4	4.7	4.8	4.1	3.4	4.0	4.7
Navy	3.8	4.9	4.8	5.8	3.4	5.2	3.9	3.6	3.8	2.6	2.8	3.6	3.4	3.9
Air Force	4.7	4.2	6.4	5.5	5.1	5.1	4.8	4.6	4.9	4.0	4.3	3.0	3.8	4.6
Marines	8.0	5.3	7.7	2.5	12.7	2.3	3.7	4.3	5.6	4.1	8.6	6.4	3.1	5.7
Total	5.1	4.6	5.7	5.1	4.9	5.2	4.5	4.2	4.6	4.0	4.0	3.4	3.7	4.5
