# Supplementary material for: Sex, military occupation and rank are associated with risk of anterior cruciate ligament injury in tactical-athletes
Source: BMJ Mil Health. 2022 Feb 14;169(6):535–41. doi: 10.1136/bmjmilitary-2021-002059 (PMC10715491; doi:10.1136/bmjmilitary-2021-002059)
Supplement: Supplementary data [file bmjmilitary-2021-002059supp003.pdf]

Supplemental Table 3: ACL injury counts, population at risk, and injury rates (per 1,000 person-years) by year for enlisted males

**Counts**	2006	2007	2008	2009	2010	2011	2012	2013	2014	2015	2016	2017	2018	Total
Army	2,141	2,243	2,377	2,385	2,553	2,505	2,399	2,293	1,970	1,640	1,495	1,301	1,064	26,366
Navy	1,399	1,239	1,249	1,236	1,167	1,134	1,029	1,057	969	856	793	668	621	13,417
Air Force	1,229	1,254	1,200	1,099	1,070	1,132	1,105	1,041	950	807	907	705	699	13,198
Marines	979	1,032	952	1,032	995	1,011	991	854	749	631	626	585	540	10,977
Total	5,748	5,768	5,778	5,752	5,785	5,782	5,524	5,245	4,638	3,934	3,821	3,259	2,924	63,958
**Population**														
Army	356,602	368,968	385,276	397,813	406,484	407,158	392,488	375,912	357,011	339,619	325,696	321,308	322,174	4,756,511
Navy	252,588	241,732	235,115	232,772	228,757	224,608	218,499	217,744	218,836	219,525	218,162	213,620	216,444	2,938,404
Air Force	219,775	212,992	207,765	211,088	213,972	214,062	214,789	215,002	209,281	200,777	202,960	207,575	209,625	2,739,662
Marines	149,900	152,982	162,916	170,257	169,126	166,945	163,060	160,424	155,635	150,625	149,883	149,024	149,423	2,050,201
Total	978,865	976,674	991,073	1,011,930	1,018,340	1,012,773	988,837	969,083	940,763	910,546	896,701	891,528	897,665	12,484,777
**Rate**														
Army	6.0	6.1	6.2	6.0	6.3	6.2	6.1	6.1	5.5	4.8	4.6	4.0	3.3	5.5
Navy	5.5	5.1	5.3	5.3	5.1	5.0	4.7	4.9	4.4	3.9	3.6	3.1	2.9	4.6
Air Force	5.6	5.9	5.8	5.2	5.0	5.3	5.1	4.8	4.5	4.0	4.5	3.4	3.3	4.8
Marines	6.5	6.7	5.8	6.1	5.9	6.1	6.1	5.3	4.8	4.2	4.2	3.9	3.6	5.4
Total	5.9	5.9	5.8	5.7	5.7	5.7	5.6	5.4	4.9	4.3	4.3	3.7	3.3	5.1
