# Supplementary material for: Sex, military occupation and rank are associated with risk of anterior cruciate ligament injury in tactical-athletes
Source: BMJ Mil Health. 2022 Feb 14;169(6):535–41. doi: 10.1136/bmjmilitary-2021-002059 (PMC10715491; doi:10.1136/bmjmilitary-2021-002059)
Supplement: Supplementary data [file bmjmilitary-2021-002059supp004.pdf]

Supplemental Table 4: ACL injury counts, population at risk, and injury rates (per 1,000 person-years) by year for enlisted females

**Counts**	2006	2007	2008	2009	2010	2011	2012	2013	2014	2015	2016	2017	2018	Total
Army	317	284	300	311	291	335	291	277	235	221	210	172	162	3,406
Navy	157	155	171	174	120	147	160	151	148	152	149	140	129	1,953
Air Force	218	227	184	209	190	157	164	174	159	148	127	150	145	2,252
Marines	55	57	55	59	74	78	54	52	63	44	45	54	49	739
Total	747	723	710	753	675	717	669	654	605	565	531	516	485	8,350
**Population**														
Army	57,078	57,448	58,491	59,505	60,574	60,858	58,291	56,108	54,367	53,170	52,769	53,278	53,860	735,798
Navy	42,281	40,962	40,877	41,868	42,966	43,936	44,216	46,119	47,615	49,165	50,548	50,825	52,944	594,325
Air Force	55,031	53,268	51,798	52,144	51,625	50,689	50,062	49,662	48,046	46,445	47,724	49,930	51,819	658,244
Marines	9,955	10,320	10,954	11,622	12,120	12,419	12,484	12,710	12,816	12,703	13,165	13,716	14,136	159,121
Total	164,345	161,998	162,120	165,140	167,286	167,902	165,054	164,600	162,844	161,484	164,207	167,749	172,759	2,147,488
**Rate**														
Army	5.6	4.9	5.1	5.2	4.8	5.5	5.0	4.9	4.3	4.2	4.0	3.2	3.0	4.6
Navy	3.7	3.8	4.2	4.2	2.8	3.3	3.6	3.3	3.1	3.1	2.9	2.8	2.4	3.3
Air Force	4.0	4.3	3.6	4.0	3.7	3.1	3.3	3.5	3.3	3.2	2.7	3.0	2.8	3.4
Marines	5.5	5.5	5.0	5.1	6.1	6.3	4.3	4.1	4.9	3.5	3.4	3.9	3.5	4.6
Total	4.5	4.5	4.4	4.6	4.0	4.3	4.1	4.0	3.7	3.5	3.2	3.1	2.8	3.9
